# 8-Meth­oxy-3-methyl-3,4-di­hydro-2*H*-1,3-benzoxazine

**DOI:** 10.1107/S1600536813026706

**Published:** 2013-10-05

**Authors:** Jing Zhu, Xiang-Xiang Yang, Long-Yu Xu, Zhi-Dong Ren, Ling-Bo Qu

**Affiliations:** a School of Chemistry and Chemical Engineering, Henan University of Technology, Zhengzhou 450001, People’s Republic of China

## Abstract

The title compound, C_10_H_13_NO_2_, crystallizes with two crystallographically independent mol­ecules of similar geometry in the asymmetric unit; the six-membered oxazine rings adopts a half-chair conformation. Neither hydrogen bonds nor π–π inter­actions are observed in the crystal structure.

## Related literature

For the synthesis and applications of 1,3-benzoxazines, see: Holly & Cope (1944 ▶); Gu *et al.* (1998 ▶); Zheng *et al.* (2011 ▶); Rimdusit & Ishida (2000 ▶); Stewart (2009 ▶); Ning & Ishida (1994 ▶). For puckering parameters, see: Cremer & Pople (1975 ▶).
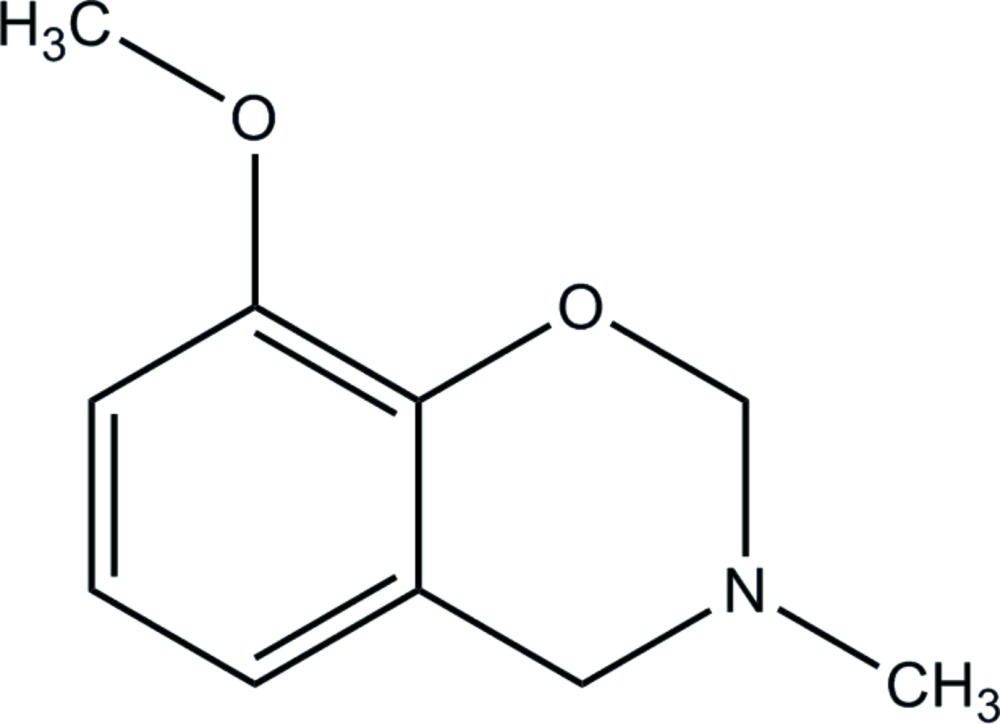


## Experimental

### 

#### Crystal data

C_10_H_13_NO_2_
*M* *_r_* = 179.21
Monoclinic, 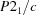

*a* = 23.4234 (14) Å
*b* = 5.0054 (3) Å
*c* = 15.9408 (10) Å
β = 97.210 (6)°
*V* = 1854.2 (2) Å^3^
*Z* = 8
Cu *K*α radiation
μ = 0.73 mm^−1^
*T* = 291 K
0.22 × 0.20 × 0.18 mm

#### Data collection

Agilent Xcalibur (Eos, Gemini) diffractometer
Absorption correction: multi-scan (*CrysAlis PRO*; Agilent, 2012 ▶) *T*_min_ = 0.783, *T*_max_ = 1.000
6857 measured reflections
3281 independent reflections
2471 reflections with *I* > 2σ(*I*)
*R*_int_ = 0.030

#### Refinement

*R*[*F* ^2^ > 2σ(*F* ^2^)] = 0.041
*wR*(*F* ^2^) = 0.114
*S* = 1.02
3281 reflections
240 parameters
H-atom parameters constrained
Δρ_max_ = 0.15 e Å^−3^
Δρ_min_ = −0.16 e Å^−3^

### 

Data collection: *CrysAlis PRO* (Agilent, 2012 ▶); cell refinement: *CrysAlis PRO*; data reduction: *CrysAlis PRO*; program(s) used to solve structure: *SHELXS97* (Sheldrick, 2008 ▶); program(s) used to refine structure: *SHELXL97* (Sheldrick, 2008 ▶); molecular graphics: *OLEX2* (Dolomanov *et al.*, 2009 ▶); software used to prepare material for publication: *OLEX2*.

## Supplementary Material

Crystal structure: contains datablock(s) I, global. DOI: 10.1107/S1600536813026706/rz5083sup1.cif

Structure factors: contains datablock(s) I. DOI: 10.1107/S1600536813026706/rz5083Isup2.hkl

Supplementary material file. DOI: 10.1107/S1600536813026706/rz5083Isup3.cml

Additional supplementary materials:  crystallographic information; 3D view; checkCIF report

## Figures and Tables

**Fig. 1.**
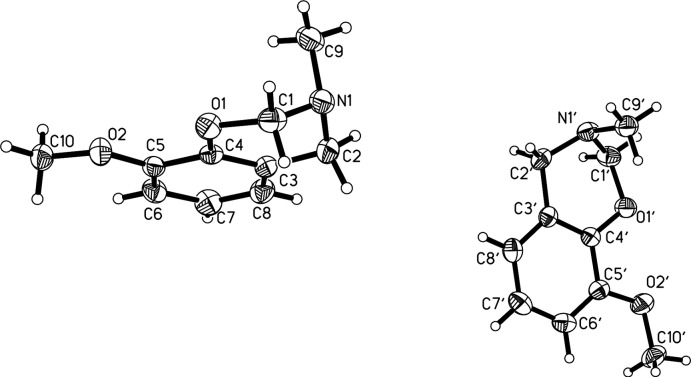
The asymmetric unit of the title complound with displacement ellipsoids drawn at the 50% probability level.

## supplementary crystallographic information

### 1. Comment

Benzo[*e*][1,3]oxazines, which are synthesized by an amine, a phenolic compound and formaldehyde *via* Mannich reaction (Holly & Cope, 1944), are a useful class of heterocyclic compounds. They can be cured *via* thermal ring-opening polymerization to construct a novel class of thermosetting resins called polybenzoxazins (Ning & Ishida, 1994). Polybenzoxazines have been widely used as automobile braking materials (Gu *et al.*, 1998), copper clad laminates (Zheng *et al.*, 2011), electronic packaging materials (Rimdusit & Ishida, 2000), aircraft cabin sidewalls (Stewart, 2009). The title compound was prepared by reaction of 2-methoxyphenol, formaldehyde and methylamine and its crystal structure is described herein.

The asymmetric unit of the title compound consists of two crystallographically independent molecules of similar geometry (Fig. 1). In both molecules the six-membered oxazine rings adopt a half-chair conformation, with atoms N1, C1 and N1', C1' displaced on opposite sides of the C2-C4/O1 (r.m.s deviation 0.0017 Å) and C2'-C4'/O1' (r.m.s deviation 0.0024 Å) mean planes by 0.4941 (15), 0.2019 (17) Å and 0.3664 (16), 0.351 (2) Å, respectively. The puckering parameters (Cremer & Pople, 1975) are *Q* = 0.4664 (16) Å, θ = 129.48 (19)°, φ = -75.7 (2)° for ring O1/C1/N1/C2–C4, and *Q* = 0.4722 (17) Å, θ = 128.97 (18)°, φ = -89.0 (3)° for ring O1'/C1'/N1'/C2'–C4'. In the crystal structure, no hydrogen bonding or π–π stacking interactions are observed.

### 2. Experimental

Methylamine (40 wt% in water; 3.9 g, 0.05 mol), formaldehyde (37% wt in water; 8.1 g, 0.1 mol), 4-methoxyphenol (6.2 g, 0.05 mol) and 1, 4-dioxine (50 ml) were added to a 250 ml flask equipped with a condenser. The mixture was stirred at 90 °C for 5 h. After condensed by rotary evaporator, a yellowish-brown viscous liquid was obtained and set at room temperature for a few hours. After washing several times with methanol, a yellowish powder was precipitated. Anhydrous ether was used to dissolve the powder, and colourless crystals suitable for X-ray diffraction analysis were obtained by slow evoporation of the solvent.

### 3. Refinement

All H atoms were refined using a riding model approximation, with C—H = 0.93–0.97 Å and with *U*_iso_(H) = 1.2 *U*_eq_(C) or 1.5 *U*_eq_(C) for methyl H atoms.

### Crystal data

**Table:**

C_10_H_13_NO_2_	*F*(000) = 768
*M**_r_* = 179.21	*D*_x_ = 1.284 Mg m^−^^3^
Monoclinic, *P*2_1_/*c*	Cu *K*α radiation, λ = 1.5418 Å
Hall symbol: -P 2ybc	Cell parameters from 2369 reflections
*a* = 23.4234 (14) Å	θ = 3.2–66.9°
*b* = 5.0054 (3) Å	µ = 0.73 mm^−^^1^
*c* = 15.9408 (10) Å	*T* = 291 K
β = 97.210 (6)°	Prism, colourless
*V* = 1854.2 (2) Å^3^	0.22 × 0.20 × 0.18 mm
*Z* = 8	

### Data collection

**Table:**

Agilent Xcalibur (Eos, Gemini) diffractometer	3281 independent reflections
Radiation source: Enhance (Cu) X-ray Source	2471 reflections with *I* > 2σ(*I*)
Graphite monochromator	*R*_int_ = 0.030
Detector resolution: 16.2312 pixels mm^-1^	θ_max_ = 67.1°, θ_min_ = 3.8°
ω scans	*h* = −19→27
Absorption correction: multi-scan (*CrysAlis PRO*; Agilent, 2012)	*k* = −3→5
*T*_min_ = 0.783, *T*_max_ = 1.000	*l* = −18→19
6857 measured reflections	

### Refinement

**Table:**

Refinement on *F*^2^	Secondary atom site location: difference Fourier map
Least-squares matrix: full	Hydrogen site location: inferred from neighbouring sites
*R*[*F*^2^ > 2σ(*F*^2^)] = 0.041	H-atom parameters constrained
*wR*(*F*^2^) = 0.114	*w* = 1/[σ^2^(*F*_o_^2^) + (0.0524*P*)^2^] where *P* = (*F*_o_^2^ + 2*F*_c_^2^)/3
*S* = 1.02	(Δ/σ)_max_ < 0.001
3281 reflections	Δρ_max_ = 0.15 e Å^−^^3^
240 parameters	Δρ_min_ = −0.16 e Å^−^^3^
0 restraints	Extinction correction: *SHELXL97* (Sheldrick, 2008), Fc^*^=kFc[1+0.001xFc^2^λ^3^/sin(2θ)]^-1/4^
Primary atom site location: structure-invariant direct methods	Extinction coefficient: 0.0041 (3)

### Special details

**Table:**

Geometry. All e.s.d.'s (except the e.s.d. in the dihedral angle between two l.s. planes) are estimated using the full covariance matrix. The cell e.s.d.'s are taken into account individually in the estimation of e.s.d.'s in distances, angles and torsion angles; correlations between e.s.d.'s in cell parameters are only used when they are defined by crystal symmetry. An approximate (isotropic) treatment of cell e.s.d.'s is used for estimating e.s.d.'s involving l.s. planes.
Refinement. Refinement of *F*^2^ against ALL reflections. The weighted *R*-factor *wR* and goodness of fit *S* are based on *F*^2^, conventional *R*-factors *R* are based on *F*, with *F* set to zero for negative *F*^2^. The threshold expression of *F*^2^ > σ(*F*^2^) is used only for calculating *R*-factors(gt) *etc*. and is not relevant to the choice of reflections for refinement. *R*-factors based on *F*^2^ are statistically about twice as large as those based on *F*, and *R*- factors based on ALL data will be even larger.

### Fractional atomic coordinates and isotropic or equivalent isotropic displacement parameters (Å^2^)

**Table:**

	*x*	*y*	*z*	*U*_iso_*/*U*_eq_	
O1	0.93754 (5)	0.7027 (2)	0.81029 (8)	0.0481 (3)	
O2	0.93639 (5)	1.0466 (3)	0.93130 (8)	0.0507 (3)	
N1	0.89328 (8)	0.5131 (3)	0.67831 (9)	0.0538 (4)	
C1	0.93598 (8)	0.4853 (3)	0.74936 (12)	0.0526 (4)	
H1A	0.9734	0.4725	0.7294	0.063*	
H1B	0.9293	0.3192	0.7779	0.063*	
C2	0.83649 (9)	0.5122 (4)	0.70753 (12)	0.0549 (5)	
H2A	0.8282	0.3343	0.7268	0.066*	
H2B	0.8075	0.5574	0.6607	0.066*	
C3	0.83340 (7)	0.7092 (3)	0.77871 (10)	0.0429 (4)	
C4	0.88444 (7)	0.7904 (3)	0.82559 (10)	0.0384 (3)	
C5	0.88378 (7)	0.9799 (3)	0.89037 (9)	0.0395 (3)	
C6	0.83181 (8)	1.0838 (4)	0.90755 (11)	0.0498 (4)	
H6	0.8309	1.2096	0.9503	0.060*	
C7	0.78089 (8)	1.0000 (4)	0.86090 (12)	0.0566 (5)	
H7	0.7460	1.0694	0.8729	0.068*	
C8	0.78165 (8)	0.8159 (4)	0.79739 (12)	0.0540 (5)	
H8	0.7472	0.7620	0.7665	0.065*	
C9	0.90299 (11)	0.7484 (4)	0.62759 (12)	0.0672 (6)	
H9A	0.8977	0.9070	0.6595	0.101*	
H9B	0.8761	0.7481	0.5769	0.101*	
H9C	0.9415	0.7445	0.6131	0.101*	
C10	0.93882 (9)	1.2585 (4)	0.99146 (12)	0.0551 (5)	
H10D	0.9778	1.2823	1.0170	0.083*	
H10E	0.9150	1.2153	1.0343	0.083*	
H10F	0.9253	1.4206	0.9635	0.083*	
O1'	0.33443 (5)	0.5282 (3)	0.32961 (8)	0.0572 (4)	
O2'	0.28466 (6)	0.1922 (3)	0.42062 (9)	0.0659 (4)	
N1'	0.41648 (7)	0.7858 (3)	0.30046 (10)	0.0520 (4)	
C1'	0.35547 (8)	0.7584 (4)	0.28753 (13)	0.0590 (5)	
H1'A	0.3430	0.7444	0.2273	0.071*	
H1'B	0.3383	0.9184	0.3078	0.071*	
C2'	0.43450 (8)	0.8338 (4)	0.39037 (12)	0.0521 (4)	
H2'A	0.4257	1.0171	0.4037	0.063*	
H2'B	0.4759	0.8107	0.4020	0.063*	
C3'	0.40554 (7)	0.6489 (3)	0.44663 (11)	0.0435 (4)	
C4'	0.35742 (7)	0.5065 (3)	0.41245 (10)	0.0426 (4)	
C5'	0.33050 (7)	0.3264 (4)	0.46242 (11)	0.0467 (4)	
C6'	0.35111 (8)	0.2984 (4)	0.54686 (12)	0.0547 (5)	
H6'	0.3334	0.1806	0.5806	0.066*	
C7'	0.39836 (9)	0.4464 (4)	0.58138 (12)	0.0622 (5)	
H7'	0.4118	0.4299	0.6386	0.075*	
C8'	0.42559 (8)	0.6174 (4)	0.53176 (12)	0.0566 (5)	
H8'	0.4577	0.7129	0.5555	0.068*	
C9'	0.44587 (9)	0.5579 (4)	0.26797 (13)	0.0611 (5)	
H9'A	0.4335	0.5381	0.2086	0.092*	
H9'B	0.4367	0.3986	0.2971	0.092*	
H9'C	0.4867	0.5870	0.2769	0.092*	
C10'	0.25623 (10)	0.0001 (5)	0.46693 (16)	0.0750 (7)	
H10A	0.2276	−0.0919	0.4292	0.113*	
H10B	0.2381	0.0892	0.5099	0.113*	
H10C	0.2839	−0.1262	0.4929	0.113*	

### Atomic displacement parameters (Å^2^)

**Table:**

	*U*^11^	*U*^22^	*U*^33^	*U*^12^	*U*^13^	*U*^23^
O1	0.0447 (6)	0.0478 (7)	0.0533 (7)	0.0041 (5)	0.0126 (5)	−0.0065 (5)
O2	0.0476 (6)	0.0544 (8)	0.0491 (7)	−0.0022 (5)	0.0019 (5)	−0.0106 (5)
N1	0.0802 (11)	0.0369 (8)	0.0467 (8)	−0.0029 (7)	0.0177 (7)	−0.0041 (6)
C1	0.0652 (11)	0.0365 (9)	0.0600 (11)	0.0048 (8)	0.0233 (9)	−0.0010 (8)
C2	0.0696 (11)	0.0432 (10)	0.0512 (10)	−0.0098 (9)	0.0047 (8)	−0.0056 (8)
C3	0.0495 (9)	0.0367 (9)	0.0425 (8)	−0.0048 (7)	0.0052 (7)	0.0024 (7)
C4	0.0429 (8)	0.0342 (8)	0.0395 (8)	0.0005 (6)	0.0107 (6)	0.0052 (6)
C5	0.0440 (8)	0.0382 (8)	0.0367 (8)	−0.0009 (6)	0.0070 (6)	0.0029 (6)
C6	0.0524 (9)	0.0531 (11)	0.0462 (9)	0.0048 (8)	0.0149 (7)	−0.0062 (8)
C7	0.0420 (9)	0.0655 (13)	0.0641 (11)	0.0074 (8)	0.0137 (8)	−0.0021 (9)
C8	0.0421 (9)	0.0594 (12)	0.0592 (11)	−0.0060 (8)	0.0018 (7)	0.0027 (9)
C9	0.1094 (18)	0.0485 (11)	0.0476 (10)	−0.0042 (11)	0.0247 (11)	0.0001 (8)
C10	0.0690 (12)	0.0457 (10)	0.0487 (10)	−0.0082 (9)	0.0007 (8)	−0.0047 (8)
O1'	0.0515 (7)	0.0706 (9)	0.0478 (7)	−0.0120 (6)	−0.0001 (5)	0.0058 (6)
O2'	0.0581 (8)	0.0701 (10)	0.0703 (9)	−0.0205 (7)	0.0115 (7)	0.0027 (7)
N1'	0.0555 (9)	0.0455 (9)	0.0569 (9)	0.0002 (7)	0.0140 (7)	0.0066 (7)
C1'	0.0571 (11)	0.0656 (13)	0.0535 (10)	0.0049 (9)	0.0038 (8)	0.0141 (9)
C2'	0.0529 (10)	0.0394 (10)	0.0649 (11)	−0.0051 (8)	0.0106 (8)	−0.0029 (8)
C3'	0.0427 (8)	0.0380 (9)	0.0500 (9)	0.0045 (7)	0.0067 (7)	−0.0041 (7)
C4'	0.0410 (8)	0.0437 (9)	0.0435 (8)	0.0047 (7)	0.0065 (6)	0.0002 (7)
C5'	0.0431 (8)	0.0435 (9)	0.0552 (9)	0.0022 (7)	0.0129 (7)	0.0000 (8)
C6'	0.0588 (10)	0.0534 (11)	0.0543 (10)	0.0077 (8)	0.0162 (8)	0.0108 (8)
C7'	0.0679 (12)	0.0727 (14)	0.0452 (10)	0.0100 (10)	0.0034 (8)	0.0042 (9)
C8'	0.0542 (10)	0.0595 (12)	0.0539 (10)	−0.0021 (9)	−0.0019 (8)	−0.0070 (9)
C9'	0.0687 (12)	0.0534 (11)	0.0647 (12)	−0.0026 (10)	0.0224 (9)	−0.0002 (9)
C10'	0.0768 (14)	0.0602 (14)	0.0950 (17)	−0.0200 (11)	0.0375 (13)	−0.0054 (12)

### Geometric parameters (Å, º)

**Table:**

O1—C1	1.456 (2)	O1'—C1'	1.451 (2)
O1—C4	1.3695 (19)	O1'—C4'	1.366 (2)
O2—C5	1.362 (2)	O2'—C5'	1.367 (2)
O2—C10	1.426 (2)	O2'—C10'	1.427 (2)
N1—C1	1.421 (3)	N1'—C1'	1.425 (2)
N1—C2	1.464 (2)	N1'—C2'	1.462 (2)
N1—C9	1.463 (2)	N1'—C9'	1.461 (2)
C1—H1A	0.9700	C1'—H1'A	0.9700
C1—H1B	0.9700	C1'—H1'B	0.9700
C2—H2A	0.9700	C2'—H2'A	0.9700
C2—H2B	0.9700	C2'—H2'B	0.9700
C2—C3	1.512 (2)	C2'—C3'	1.508 (2)
C3—C4	1.389 (2)	C3'—C4'	1.386 (2)
C3—C8	1.391 (2)	C3'—C8'	1.388 (3)
C4—C5	1.404 (2)	C4'—C5'	1.404 (2)
C5—C6	1.382 (2)	C5'—C6'	1.379 (3)
C6—H6	0.9300	C6'—H6'	0.9300
C6—C7	1.389 (3)	C6'—C7'	1.387 (3)
C7—H7	0.9300	C7'—H7'	0.9300
C7—C8	1.370 (3)	C7'—C8'	1.375 (3)
C8—H8	0.9300	C8'—H8'	0.9300
C9—H9A	0.9600	C9'—H9'A	0.9600
C9—H9B	0.9600	C9'—H9'B	0.9600
C9—H9C	0.9600	C9'—H9'C	0.9600
C10—H10D	0.9600	C10'—H10A	0.9600
C10—H10E	0.9600	C10'—H10B	0.9600
C10—H10F	0.9600	C10'—H10C	0.9600
			
C4—O1—C1	114.28 (13)	C4'—O1'—C1'	113.24 (14)
C5—O2—C10	117.51 (14)	C5'—O2'—C10'	117.90 (17)
C1—N1—C2	108.85 (14)	C1'—N1'—C2'	108.58 (15)
C1—N1—C9	112.13 (16)	C1'—N1'—C9'	112.47 (16)
C9—N1—C2	112.88 (17)	C9'—N1'—C2'	112.64 (15)
O1—C1—H1A	108.6	O1'—C1'—H1'A	108.8
O1—C1—H1B	108.6	O1'—C1'—H1'B	108.8
N1—C1—O1	114.52 (14)	N1'—C1'—O1'	113.74 (15)
N1—C1—H1A	108.6	N1'—C1'—H1'A	108.8
N1—C1—H1B	108.6	N1'—C1'—H1'B	108.8
H1A—C1—H1B	107.6	H1'A—C1'—H1'B	107.7
N1—C2—H2A	109.3	N1'—C2'—H2'A	109.0
N1—C2—H2B	109.3	N1'—C2'—H2'B	109.0
N1—C2—C3	111.61 (15)	N1'—C2'—C3'	112.71 (14)
H2A—C2—H2B	108.0	H2'A—C2'—H2'B	107.8
C3—C2—H2A	109.3	C3'—C2'—H2'A	109.0
C3—C2—H2B	109.3	C3'—C2'—H2'B	109.0
C4—C3—C2	118.41 (16)	C4'—C3'—C2'	119.09 (15)
C4—C3—C8	119.13 (16)	C4'—C3'—C8'	118.99 (17)
C8—C3—C2	122.45 (16)	C8'—C3'—C2'	121.92 (16)
O1—C4—C3	123.36 (15)	O1'—C4'—C3'	122.78 (16)
O1—C4—C5	116.23 (14)	O1'—C4'—C5'	116.67 (15)
C3—C4—C5	120.36 (15)	C3'—C4'—C5'	120.54 (16)
O2—C5—C4	115.17 (14)	O2'—C5'—C4'	114.85 (16)
O2—C5—C6	125.41 (15)	O2'—C5'—C6'	125.68 (17)
C6—C5—C4	119.42 (15)	C6'—C5'—C4'	119.48 (17)
C5—C6—H6	120.0	C5'—C6'—H6'	120.1
C5—C6—C7	119.92 (16)	C5'—C6'—C7'	119.81 (18)
C7—C6—H6	120.0	C7'—C6'—H6'	120.1
C6—C7—H7	119.7	C6'—C7'—H7'	119.7
C8—C7—C6	120.57 (17)	C8'—C7'—C6'	120.59 (18)
C8—C7—H7	119.7	C8'—C7'—H7'	119.7
C3—C8—H8	119.7	C3'—C8'—H8'	119.7
C7—C8—C3	120.59 (16)	C7'—C8'—C3'	120.55 (18)
C7—C8—H8	119.7	C7'—C8'—H8'	119.7
N1—C9—H9A	109.5	N1'—C9'—H9'A	109.5
N1—C9—H9B	109.5	N1'—C9'—H9'B	109.5
N1—C9—H9C	109.5	N1'—C9'—H9'C	109.5
H9A—C9—H9B	109.5	H9'A—C9'—H9'B	109.5
H9A—C9—H9C	109.5	H9'A—C9'—H9'C	109.5
H9B—C9—H9C	109.5	H9'B—C9'—H9'C	109.5
O2—C10—H10D	109.5	O2'—C10'—H10A	109.5
O2—C10—H10E	109.5	O2'—C10'—H10B	109.5
O2—C10—H10F	109.5	O2'—C10'—H10C	109.5
H10D—C10—H10E	109.5	H10A—C10'—H10B	109.5
H10D—C10—H10F	109.5	H10A—C10'—H10C	109.5
H10E—C10—H10F	109.5	H10B—C10'—H10C	109.5
			
O1—C4—C5—O2	−1.7 (2)	O1'—C4'—C5'—O2'	0.8 (2)
O1—C4—C5—C6	177.98 (15)	O1'—C4'—C5'—C6'	−179.01 (16)
O2—C5—C6—C7	179.74 (17)	O2'—C5'—C6'—C7'	179.68 (18)
N1—C2—C3—C4	−21.6 (2)	N1'—C2'—C3'—C4'	−15.2 (2)
N1—C2—C3—C8	156.76 (17)	N1'—C2'—C3'—C8'	164.96 (17)
C1—O1—C4—C3	−9.1 (2)	C1'—O1'—C4'—C3'	−14.7 (2)
C1—O1—C4—C5	173.47 (14)	C1'—O1'—C4'—C5'	166.48 (16)
C1—N1—C2—C3	50.6 (2)	C1'—N1'—C2'—C3'	45.4 (2)
C2—N1—C1—O1	−62.57 (19)	C2'—N1'—C1'—O1'	−64.2 (2)
C2—C3—C4—O1	0.5 (2)	C2'—C3'—C4'—O1'	−0.7 (2)
C2—C3—C4—C5	177.80 (15)	C2'—C3'—C4'—C5'	178.01 (16)
C2—C3—C8—C7	−178.06 (18)	C2'—C3'—C8'—C7'	−179.74 (18)
C3—C4—C5—O2	−179.24 (14)	C3'—C4'—C5'—O2'	−177.96 (15)
C3—C4—C5—C6	0.5 (2)	C3'—C4'—C5'—C6'	2.2 (3)
C4—O1—C1—N1	41.2 (2)	C4'—O1'—C1'—N1'	48.5 (2)
C4—C3—C8—C7	0.3 (3)	C4'—C3'—C8'—C7'	0.4 (3)
C4—C5—C6—C7	0.1 (3)	C4'—C5'—C6'—C7'	−0.5 (3)
C5—C6—C7—C8	−0.4 (3)	C5'—C6'—C7'—C8'	−1.2 (3)
C6—C7—C8—C3	0.2 (3)	C6'—C7'—C8'—C3'	1.3 (3)
C8—C3—C4—O1	−178.00 (15)	C8'—C3'—C4'—O1'	179.13 (16)
C8—C3—C4—C5	−0.7 (2)	C8'—C3'—C4'—C5'	−2.1 (3)
C9—N1—C1—O1	63.0 (2)	C9'—N1'—C1'—O1'	61.2 (2)
C9—N1—C2—C3	−74.6 (2)	C9'—N1'—C2'—C3'	−79.85 (19)
C10—O2—C5—C4	173.49 (14)	C10'—O2'—C5'—C4'	178.22 (17)
C10—O2—C5—C6	−6.2 (2)	C10'—O2'—C5'—C6'	−1.9 (3)
